# Experiments upon the Effects of High Degrees of Heat upon the Animal Economy

**Published:** 1810-04

**Authors:** F. E. Delaroche


### *Experiments upon the Effects of high Degrees of Heat upon the Animal Economy.* By F. E. Delaroche.

( Journal de Physique, tom. 63—1809. )

PHYSIOLOGISTS have been much engaged with a consideration of the high degrees of heat capable of being supported by man as well as what it produces upon animals. The very interesting experiments of Drs. Fordyce and Blagden leave us on this subject but little to desire. I have thought it right, however, to repeat some of these experiments, with a view to ascertain whether other individuals could support this degree of temperature equally as these naturalists. With this intention, M. Berger and myself were exposed in a stove of different degrees of heat, our bodies being naked and guarded from the heat of the furnace by a screen of silk. We calculated the temperature of the place, by means of a thermometer attached to the wall, $4\frac{1}{2}$ feet from the ceiling.

Temperature of stove at beginning of the experiment, 70°
At the conclusion . . . . . . . . . . . . . . . 72
I entered the stove at . . . . . . . . . . . . $5^h$ 17′
Weight of my body at the time of entering 128lb. 11oz. 3gr.
10′ after coming out . . . . . . . . 128lb. 5oz. 0gr.

I perceived

I perceived very sensibly on entering, the impression of the warm air, but without being incommoded in the first moments. At 3$^h$ 21′, some small drops of sweat appeared upon my brow. At 3$^h$ 22′, the whole of my body was enveloped in an abundant perspiration. I began to experience at this time a slight weakness, and some distress during respiration, which continued to increase. I was obliged to withdraw.

### Experiment II.

Temperature at the beginning of the experiment . 72°
At the conclusion . . . . . . . . . . . . . . . 70
M. Berger entered at . . . . . . . . . . . . . 3$^h$ 41′
He quitted it at . . . . . . . . . . . . . . . 3 54
Weight of his body at the commencement . 51,460 grs.
At the conclusion, and after being 5′ out . 51,145 grs.

He experienced, on entering, a slight degree of burning about the nostrils and nipples. The sweat which began to appear upon his forehead, at 3$^h$ 45′, flowed two minutes afterwards, in abundance, from every part of the body. At the moment of his quitting the stove, he experienced a slight degree of weakness, and even a tendency to fall down. His pulse an instant before, beat 128 times in a minute.

### Experiment III.

Temperature of the stove at the beginning of the experiment . . . . . . . . . . . . . . . . . 87½°
At the end . . . . . . . . . . . . . . . . . 86
M. Berger entered the stove at . . . . . . . 4$^h$ 32′
And departed at . . . . . . . . . . . . . . . 39
Weight on his entering . 50890 grs. 103lb. 15oz. 3grs.
Two minutes afterwards . 50670 grs. 103lb. 8oz. 1½ grs.

He experienced a very considerable burning sensation about his nipples, at his nostrils; and even over the whole of his face His body was suffused by perspiration at 4$^h$ 36′ in great abundance. When he came out, he was weak, and much indisposed. A moment before, he could not count the frequency of his pulse. Three quarters of an hour after he came out, he was restored to his natural state.

These experiments perfectly well accord with those which are recorded by Dr. Blagden, inasmuch as that man can for a short time maintain a degree of temperature very considerably above the ordinary temperature of the medium um in which he lives; but they also prove that there is a great difference between different individuals, relatively to the extent of this faculty. In all of these experiments, M. Berger supported the experiments much better than myself, as may be perceived by a comparison of the experiments. If on the other hand, these be compared with the experiments of the English physicians, we shall perceive that these gentlemen, or at least *Dr. Blagden,* suffered much less than *M. Berger* and myself. In fact, Dr. Blagden did in one experiment, bear for eight minutes, a temperature which was between 240° and 250° Fahrenheit, (92¼ and 102⅓° Reaumur,) and was not afterwards at all the worse for it; and that M. Berger was scarcely in the stove the space of seven minutes, at the temperature of 87° Reaumur. On another trial, Dr. Blagden supported a degree of heat during 12′ of 222° Fahrenreit, (83⅓ Reaumur,) without experiencing any inconvenience beyond fatigue.

The author has made a great number of experiments upon the degrees of heat which animals and man can support in warm baths and in vapour baths; on the effect which is exercised by heat upon respiration; upon the relation which exists between evaporation and the matter of transpiration, and the faculty possessed by animals of producing cold; on the influence which heat exerts upon respiration; and upon the state of the body in animals which have died in consequence of excess of heat.

He concludes his researches by the following observations.

Such are the researches which I have made to ascertain the effects which are produced by a high degree of heat upon man and animals. I could wish to give them more publicity, and to render them more complete, but the time which this labour would cost, and the difficulty of the subject itself, have not permitted me to accomplish this. Although I cannot deduce general consequences, I proceed, notwithstanding, to recapitulate, in a few words, the results which they have furnished to me.

1. Their first experiments have in view to determine what is the degree of heat necessary, to cause animals to perish. They have shewn me that exposure to a temperature of 50°, and even of 45° Reaumur, were sufficient to cause death in animals of small size. It is even probable, that a less intense heat, but the action of which is longer continued, may produce this effect.

2. M. *Berger* and myself, have stated by experiments, made made upon ourselves, the power man possesses of supporting, though, in truth, but for a very short space of time, exposure to high temperatures. The comparison of these experiments, whether with each other, or with those of Dr. Blagden, has demonstrated that there may be a great difference between different individuals relatively to the extent of this faculty.

3. Those experiments wherein we were exposed to the action of aqueous vapours, have proved to us the truth of the assertion of Dr. *Fordyce*, viz. that the impression of an air charged with vapours, is at an equal degree of temperature, much more intolerable than dry air.

4. We have been anxious precisely to estimate, by the help of the balance, the effects of heat upon the transpiration. The loss of weight which we experience by this means, appears to be in a direct proportion of the elevation of temperature to which we have been exposed. We have also ascertained that the heat of the aqueous vapour, excited exudation with much more energy than a dry heat.

5. I believe that I have demonstrated that the faculty, enjoyed by man and animals, of maintaining an equable temperature, although exposed to a high degree of heat, is much less extensive than is generally believed, according to the experiments of M. M. *Blagden* and *Fordyce*; and that it is in no wise comparable to the faculty, which they have of resisting cold, in maintaining a temperature above that of the ambient medium.

6. Although this faculty should be restrained, it would nevertheless exist. It is then interesting to ascertain the cause. Does it merely render in the refrigeration, produced by evaporation, as some physiologists think? I am not in a condition to answer whether the same thing takes place with respect to cold blooded animals. I can merely state, that inanimate bodies, the inner surface of which is moist and susceptible of evaporation, would acquire a less elevated temperature, when exposed to a high degree of heat, than cold blooded animals placed under the same circumstances.

7. I have also endeavoured to determine what is the influence of heat upon the phænomena of respiration. Dr. *Crawford*, who occupied himself much upon this subject, believed that the deterioration of the air, by the respiration of animals, was by so much the less as the heat to which they were exposed was greater. In sufficiently numerous experiments, made by myself, I have not perceived any any fixed proportion between the degree of deterioration in which animals have been confined, and that of the temperature to which they have been exposed.

8. I, lastly, examined the circumstances which accompanied death, occasioned by exposure to heat; for I in particular, examined the state of the body of animals who died in this manner. The phænomena which anatomical inspection has presented, among which, the most remarkable, is the dim motion of muscular irritability, have not been sufficiently constant to permit me to draw any conclusion as to the cause of this death.

